# An Analysis of the Spatial Characteristics and Transport Fluxes of BTEX in Soil and Atmospheric Phases at a Decommissioned Steel Mill Site in China with a Long History

**DOI:** 10.3390/toxics11100868

**Published:** 2023-10-18

**Authors:** Xuwei Li, Wenyi Xie, Da Ding, Mengjie Wang, Lingya Kong, Dengdeng Jiang, Shaopo Deng

**Affiliations:** 1 Nanjing Institute of Environmental Sciences, Ministry of Ecology and Environment of China, Nanjing 210042, China; lixuwei@nies.org (X.L.); xiewenyi@nies.org (W.X.); dingda@nies.org (D.D.); wangmengjie@nies.org (M.W.); kly@nies.org (L.K.); jiangdengdeng@nies.org (D.J.); 2 State Environmental Protection Key Laboratory of Soil Environmental Management and Pollution Control, Nanjing 210042, China

**Keywords:** correlation, transport flux, non-carcinogenic risk, carcinogenic risk

## Abstract

BTEX (benzene, toluene, ethylbenzene, and xylene), as characteristic pollutants in chemical plant sites, are widely present in the environment and pose a serious threat to the health and safety of nearby residents. Studying the spatial distribution characteristics and transport fluxes of BTEX in soil and air at contaminated sites and the health risks they pose to humans is of great significance for fine pollution control and environmental management. This study took a typical decommissioned steel plant as a case study. A total of 23 soil and air samples were collected from different locations to investigate the spatial distribution characteristics of BTEX in soil and air. The transport and fate of BTEX in soil and air were evaluated using the fugacity model, and finally, a human health risk assessment was conducted. The results indicate a relatively severe level of benzene pollution in both soil and air. The maximum exceedance factor of benzene in soil samples is 31.5, with the concentration exceedance depth at 1.5 m. The maximum concentration of benzene in air samples is 4.98 μg·m^−3^. Benzene, at 5.9% of the site, shows a low flux with negative values, while other components at various locations all exhibit a trend of transport from the soil phase to the atmospheric phase. Benzene is the pollutant that contributes the most to the transport flux from soil to air within the site. The coking area and sewage treatment area are key areas within the steel mill where BTEX accumulate easily in the soil. The non-carcinogenic risk values of the individual components of BTEX in the soil are below the acceptable risk level. However, the carcinogenic risk value of benzene in the children’s exposure scenario exceeds the carcinogenic risk level of 10^−6^. The carcinogenic risk range of various components of BTEX in the air is 2.63 × 10^−6^~3.88 × 10^−5^, with 28.6% of the locations exceeding the threshold of 10^−6^. The range of the total HI (hazard index) is 2.08 × 10^−4^~1.81 × 10^−1^, all of which is below the safety threshold of 1. The results of this study will provide scientific support for the fine pollution control and environmental management of industrial contaminated sites with BTEX as their typical pollutants.

## 1. Introduction

The rapid progress of urbanization in China, in combination with an enormous spike in the market demand for land transfer, has resulted in the abandonment of a large number of industrial enterprises. However, these sites often become sources of soil and/or air pollution, thereby posing a threat to the health and safety of the living environment around them [1,2,3]. BTEX, as one of the most typical pollutants commonly found in chemical plant sites, are commonly found in such environments. BTEX are typically used in industries such as petroleum, chemical, and coking. Its representative substances include Benzene, Toluene, Ethylbenzene, and xylene [4]. Of the above substances, the International Agency for Research on Cancer (IARC) has confirmed Benzene to be a carcinogen that poses a significant risk to human health [5]. Studies have also shown that toluene, ethylbenzene, and xylene have paralytic and stimulant effects on the central nervous system [6,7]. In the background of these studies and discoveries, there have been numerous reports on the health risks associated with BTEX. Garg et al. found that the carcinogenic risk of BTEX in the ambient air of Delhi, India, ranged from 4.09 × 10^−6^ to 3.40 × 10^−5^ (within a 95% confidence interval), which is in excess of the acceptable value of 1.0 × 10^−6^ [8]. The study conducted by Miri et al. indicated that the lifetime cancer risk (LTCR) posed by inhalation of benzene had an average value of 3.93 × 10^−7^, lower than the limits recommended by the United States Environmental Protection Agency (USEPA) and the World Health Organization (WHO) [9]. The HQ (Hazard Quotient), which represents the non-carcinogenic risk index, for BTEX compounds was less than 1. Studies on soil media also showed higher risks for residential land and subsoil [10,11]. BTEX, especially benzene and toluene, exhibit a migratory behavior in environmental media [12]. After entering the environment through pathways such as air disposition and industrial utilization, they can be released into the air and the surrounding environment [13]. Therefore, the transport and fate of these compounds, especially upon release into the environmental media, are becoming environmental concerns that are currently receiving great attention.

Accurately evaluating and assessing the presence of BTEX in contaminated sites, with particular attention to pollution patterns and characteristics, the health risks posed, and their transport patterns, is a significant step forward in the efforts to control, manage, and properly account for these sites. Currently, research both domestically and internationally on BTEX in environmental media mainly focuses on indoor soil column transport experiments, numerical simulations [14,15], distribution characteristics [16], risk assessment [8,17], optimization of testing methods [18,19], and environmental behavior [20], among others. Overall, there is still a lack of conclusive and reliable studies on the transport and fate of BTEX in both soil and air media in actual field applications. Many researchers have conducted studies using gas flux chambers to investigate and simulate the volatile flux of VOCs (Volatile Organic Compounds) in contaminated sites [21,22]. Ping et al., for example, conducted a study on the volatile flux of benzene in sandy soil and black soil using a flux chamber [23]. However, considering factors such as the actual site area, soil physicochemical properties, and meteorological conditions, it is difficult to achieve universality through simulated inference from chamber experiments alone.

In recent years, with the rapid development of the chemical industry in China, the impact of chemicals entering the environmental system on the ecological environment and the associated risks to human health have garnered increasing attention. The Multi-Media Pollution Model has risen to prominence as an ideal model to effectively assess the transport, transformation, and risks in the environmental system. Of all these models, none have been widely promoted in practical applications other than the Fugacity model, which has been widely used, particularly in large-scale regions, multi-phase media, and persistent toxic pollutants [24,25,26,27,28]. However, there is a lack of research which aims to actively combine various conditions such as contaminated sites, soil–air two-phase media, and volatile pollutants. Considering that many industrial polluted sites in China have complex pollutant types and diverse environmental conditions, there is a relatively limited amount of applied research on pollutant transport flux in actual field sites. This study takes a typical decommissioned steel plant site as an example and selects four functional zones, which show characteristic pollutants of BTEX, as its research areas [29]. The study analyzes the pollution characteristics of BTEX and explores their spatial distribution in soil and air, environmental fate, and the human health risks they pose. The aim is to provide scientific support for the refined control and environmental management of BTEX-contaminated sites.

## 2. Materials and Methods

### 2.1. Research Areas

The site was established in 1958, and occupies an area of approximately 2 km^2^ (or 3000 mu). After a production period of 60 years, the steel plant went out of production in 2015. The site covers a series of supporting smelting processes, including sintering, coking, ironmaking, steelmaking, and rolling. The main raw materials and intermediate products include coal tar, crude benzene, ammonia, asphalt, industrial naphthalene, sulfur, iron ore, coke, limestone, and so on. For this study, a number of areas with typical BTEX pollution characteristics were selected, including the coking area (67,957 m^2^), chemical production workshop area (38,297 m^2^), crude benzene storage tank area (9209 m^2^), and circulating water and sewage treatment area (79,784 m^2^).

Since the buildings have all been demolished, the site now lies vacant. Geological exploration indicates that the soil layers from top to bottom are as follows: (1) miscellaneous fill layer, mainly composed of cohesive soil, with a thickness ranging from 1.20 to 6.50 m; (2) clay layer, with a thickness ranging from 2.70 to 21.50 m and a permeability coefficient of 1.0 × 10^−6^ cm·s^−1^; and (3) silty clay layer, with an exposed thickness ranging from 1.50 to 13.20 m and a permeability coefficient of 1.0 × 10^−5^ cm·s^−1^. The fluctuation of the groundwater level is greatly influenced by atmospheric precipitation, with an annual amplitude depth of approximately 1.50 m.

### 2.2. Sample Collection and Methods of Analysis

**Figure 1:**
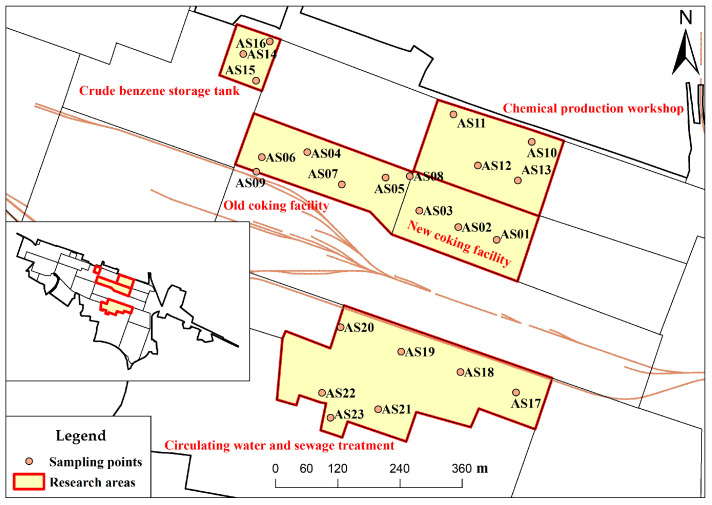

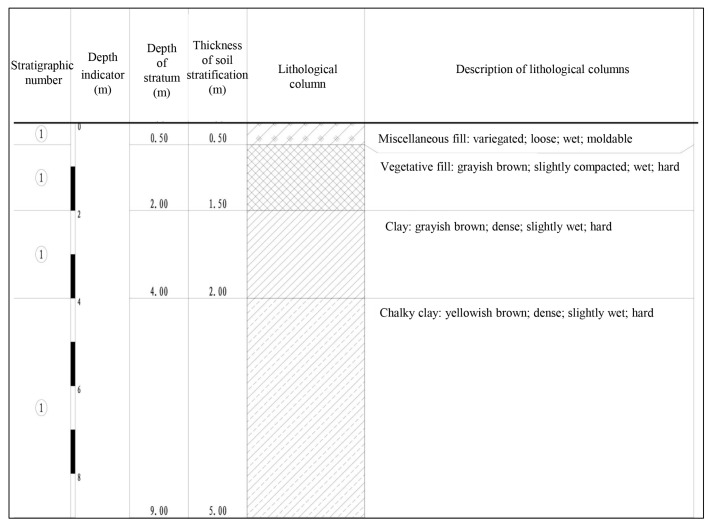
Distribution of sampling sites and lithological column.

In accordance with the “Technical Guidelines for Monitoring During Risk Control and Remediation of Soil Contamination of Land for Construction” (HJ25.2-2019) [30], the survey sampling was conducted using a combination of systematic grid layout and judgment-based density placement. A total of 23 soil and air sampling points were established as shown in Figure 1. At each soil sampling point, samples were collected separately from the vadose zone (0~0.5 m), middle, and bottom layers. The soil sampling method followed the requirements outlined in the “Technical Specifications for Soil Environmental Monitoring” (HJ/T166-2004). Air samples were collected using Summa canisters at a height of 1.2 m and a flow rate of 0.02 L/min over 24 h.

For quantitative analysis of BTEX in soil, the headspace gas chromatography-mass spectrometry (HS-GC-MS) method was used. The determination method referred to the “Soil and Sediment-Determination of Volatile Organic Compounds -Purge and Trap Gas Chromatography/Mass Spectrometry Method” (HJ 605-2011) [31]. The temperature program for column heating was set as follows: the initial column temperature was 50 °C, held for 3 min, and then ramped up at a rate of 6 °C·min^−1^ to 75 °C, with a solvent delay time of 1.7 min. High-purity helium gas (99.999%) was used as the carrier gas.

For the analysis of BTEX in air, the “Ambient air—Determination of volatile organic compounds—Sorbent adsorption and thermal desorption/gas chromatography mass spectrometry method” (HJ 644-2013) was employed [32]. First, a 400 mL sample was concentrated using a three-level cold trap, followed by rapid heating for desorption and introduction into the gas chromatography-mass spectrometry (GC-MS) instrument. After separation via gas chromatography, qualitative analysis was performed by comparing with the standard mass spectra and retaining the time. Quantitative analysis was then carried out using an external standard method.

### 2.3. Correlation Analysis Method

In this study, the correlation analysis method was used to analyze the correlation of five BTEX between the air and the soil. The correlation coefficient is a statistical measure used to describe the linear relationship and direction between two variables, usually represented by “r”. The specific formula is as follows in Equation (1):

(1) $$r_{\mathrm{xy}}=\frac{\sum_{i=1}^{n}\left[\left(x_{i}-\bar{x}\right)\left(y_{i}-\bar{y}\right)\right]}{\sqrt{\sum_{i=1}^{n}\left[{\left(x_{i}-\bar{x}\right)}^{2}{\left(y_{i}-\bar{y}\right)}^{2}\right]}}$$

In the equation provided, r_xy_ represents the correlation coefficient between variables x and y; n is the sample size; and x and y represent the mean values of variables x and y, respectively.

### 2.4. Construction of the Fugacity Model

In this study, a multi-media fugacity model was used to combine various environmental parameters and migration parameters in order to establish the mass balance equation between different environmental media (including air and soil) in the actual contaminated site. If the fugacity is unequal, the pollutant will migrate from the medium with higher fugacity to the one with lower fugacity, and the flux of pollutants in each environmental phase is calculated using an equation. According to the distribution mode and migration and transformation law of chemical pollutants in environmental media, the multi-media fugacity model can be divided into the Level I, II, III, and IV models [33,34]. Among them, the Level III model represents a non-equilibrium, steady-state, and mobile system, which can effectively reflect the distribution and migration of pollutants in both phases of the environmental media, and is more effectively applied to the actual environment of soil–gas exchange in this study area. The equations for Z-values and D-values between the media are listed below, with subscripts indicating the following: 1 for air, 2 for water, and 3 for soil.

Calculation of Z-value for air phase using Equations (2)–(4):

Z_11_ = 1/RT (2)

Z_13_ = 6 × 10^6^ Z_11_/P_Ls_ (3)

Z_1_ = Z_11_ + V_13_Z_13_ (4)

In the equations provided, Z_11_ is the atmospheric subphase in air, expressed in mol·m^−3^·Pa^−1^; Z_13_ is the aerosol subphase in air, expressed in mol·m^−3^·Pa^−1^; and V_13_ is the volume fraction of particles in the air.

Calculation of Z-value for soil phase using Equations (5)–(9):

Z_31_ = 1/RT (5)

H = P_L_M/S (6)

Z_32_ = 1/H (7)

Z_33_ = 0.41 × K_OW_ × y_OC_ × Z_32_(ρ_S_/1000) (8)

Z_3_ = V_31_Z_31_ + V_32_Z_32_ + V_33_Z_33_ (9)

In the equations provided, Z_31_ represents the atmospheric subphase in the soil, expressed in mol·m^−3^·Pa^−1^; Z_32_ represents the aqueous subphase in the soil, expressed in mol·m^−3^·Pa^−1^; Z_33_ represents the solid particle subphase in the soil, expressed in mol·m^−3^·Pa^−1^; H represents Henry’s law constant, expressed in Pa·m^3^·mol^−1^; P_L_ represents the vapor pressure at 25 °C, expressed in Pa; M represents the molecular weight of the pollutant, expressed in g·mol^−1^; S represents the solubility of the pollutant in water, expressed in mol·m^−3^; and K_OW_ represents the octanol–water partition coefficient.

Calculation of D-value for air-to-soil transport using Equations (10)–(14):

D_S_ = 1/(1/K_SA_A_13_Z_11_ + Y_3_/(A_13_(B_A3_Z_11_ + B_W3_Z_32_)) (10)

D_QS_ = A_12_U_Q_Z_22_ (11)

D_DS_ = A_13_U_Q_QV_13_Z_13_ (12)

D_PS_ = A_13_U_P_V_13_Z_13_ (13)

D_13_ = D_S_ + D_QS_ + D_DS_ + D_PS_ (14)

In the equations provided, D_S_ represents the diffusion process value, expressed in mol·Pa^−1^·h^−1^; D_QS_ represents the dissolution process due to rainfall, expressed in mol·Pa^−1^·h^−1^; D_DS_ represents the wet deposition value, expressed in mol·Pa^−1^·h^−1^; and D_PS_ represents the dry deposition value, expressed in mol·Pa^−1^·h^−1^.

Calculation formulae for migration flux using Equations (15)–(20):

D-value for air-to-soil transport (mol·Pa^−1^·h^−1^): D_31_ = D_S_ (15)

Fugacity of air phase (Pa): ƒ_1_ = C_1_/Z_1_ (16)

Fugacity of soil phase (Pa): ƒ_3_ = C_3_/Z_3_ (17)

Transport flux from air to soil (mol·h^−1^): N_13_ = D_13_(ƒ_1_ − ƒ_3_) (18)

Transport flux from soil to air (mol·h^−1^): N_31_ = D_31_(ƒ_3_ − ƒ_1_) (19)

Fugacity entropy: log(f_3_/f_1_) (20)

The fugacity model mainly involves thermodynamic quantities related to temperature, such as water solubility (S, g·L^−1^), vapor pressure (P, Pa) or Henry’s law constant (H, Pa·m^3^·mol^−1^), and the octanol–water partition coefficient (K_OW_, dimensionless). These parameters are associated with the distribution process of chemicals. The physicochemical parameters of the target pollutants in this study were obtained from environmental handbooks or predictive software. The parameters M, P_L_, K_OW_, and S used in the model were primarily obtained from environmental handbooks [35,36]. P_LS_ was calculated by referring to Antoine constants [36]. Environmental and migration parameters were mainly obtained from foreign scientific publications [37], including R, V_13_, V_31_, V_32_, V_33_, K_SA_, Y_3_, B_A3_, B_W3_, Q, U_P_, and other parameters. Please refer to the Supplementary Materials for the main physicochemical, environmental, and migration parameters of BTEX in this study.

### 2.5. Human Health Risk Assessment Methods

#### 2.5.1. Soil Pollution Health Risk Assessment Method

In this study, a soil pollution health risk assessment model was used to quantify the carcinogenic and non-carcinogenic risks to human health [38]. This method combines guidelines from the United States Environmental Protection Agency (USEPA) and exposure factor handbooks with the assessment model in the “Technical Guidelines for Risk Assessment of Soil Contamination of Land for Construction” (HJ25.3-2019). It also considers the health risks caused by the intake of pollutants by humans through exposure pathways such as ingestion, inhalation, and dermal contact. Some parameters in the assessment model were determined based on the specific and particular conditions at the actual site. These parameters include PM_10_, the level of concentration of pollutants in the soil, thickness of the contaminated soil layer, the physicochemical properties of the soil, and other characteristic parameters. The physicochemical and toxicological characteristic parameters of the pollutants, as well as the human exposure parameters, were referenced from the recommended values or default values in the “Technical Guidelines for Risk Assessment of Soil Contamination of Land for Construction” (HJ25.3-2019). In this study, the criteria for determining whether there is a risk to human health is that the acceptable carcinogenic risk for a single pollutant is 10^−6^, and the acceptable non-carcinogenic hazard quotient is 1 [39]. The actual values used in the exposure assessment are shown in Table 1.

#### 2.5.2. Air Pollution Health Risk Assessment Method

The risk assessment method used in this study is based on an internationally recognized health risk assessment approach, specifically the four-step process proposed by the National Academy of Sciences (NAS) in the United States, which includes hazard identification, estimation of the effect of harmful factor concentrations, and a health risk assessment [40]. The dose–response relationship data adopted in this study are recommended values from the Integrated Risk Information System (IRIS) database of the United States Environmental Protection Agency (EPA) [41], while parameters such as exposure frequency, exposure duration, and average time are referenced from the relevant literature [42,43]. When calculating the health risks associated with multiple pollutants, non-carcinogenic hazard quotients need to be summed. Currently, the non-carcinogenic risk is evaluated according to the standards set by the USEPA, where a hazard index of 1 is considered acceptable. Referenced from the health thresholds identified by the NAS, 10^−6^ is an acceptable carcinogenic risk level. Carcinogenic risks can be further divided into four risk levels: negligible risk (Risk < 1 × 10^−6^), low probability risk (1 × 10^−6^ < Risk < 1 × 10^−5^), high probability risk (1 × 10^−5^ < Risk < 1 × 10^−4^), and risk with certainty (Risk > 1 × 10^−4^).

Exposure concentrations are calculated as follows:

EC = (CA × ET × EF × ED)/(ED·365(d·a^−1^)·24(h·d^−1^)) (21)

Calculation of the non-carcinogenic hazard quotient:

HQ = EC/(RFC × 1000) (22)

Calculation of the hazard index:

(23) HI=∑HQ

Calculation of the carcinogenic risk:

Risk = EC × IUR (24)

In the equations provided, EC is the exposure concentration, expressed in μg·m^−3^; CA is the measured concentration, expressed in μg·m^−3^; ET is the exposure time, expressed in h·d^−1^; EF is the exposure frequency, expressed in d·a^−1^; ED is the exposure duration, expressed in a; RfC is the reference dose for the pollutant, expressed in mg·m^−3^; IUR is the unit risk for inhalation carcinogens, expressed in μg^−1^·m^3^; Risk is the carcinogenic risk; and HI is the hazard index.

## 3. Results and Discussion

### 3.1. Spatial Distribution Characteristics of BTEX

#### 3.1.1. Pollution Characteristics and Spatial Distribution of BTEX in Soil

**Figure 2:**
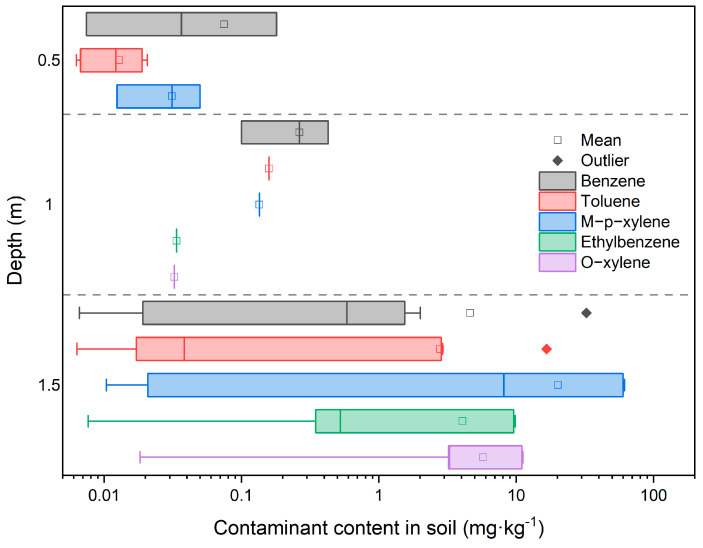
Vertical distribution of soil BTEX pollution at sampling points in the site.

Upon entry into the soil, pollutants are influenced by the physicochemical properties of the soil, pollutant characteristics, soil adsorption, etc. A combination of these factors results in certain characteristics in terms of concentration and spatial distribution. From the analysis of the BTEX detection in soil samples shown in Table 2, the detection rates are as follows: ethylbenzene and *o*-xylene < *m*/*p*-xylene < benzene and toluene. The detection rate for benzene and toluene in samples is both 30.43%. Malakootian et al. monitored BTEX at 30 independent locations in Zarand, Kermam, southeastern Iran. The order of the detection rates for BTEX were: benzene < ethylbenzene < *o*-xylene < *m*/*p*-xylene < toluene [44]. Abdel-Rahman et al. also demonstrated in their study that benzene was one of the most detected and excessive pollutants in soil samples at their site. This suggests that benzene and toluene are more widely distributed in chemical plant sites [45]. Only benzene exceeded the screening value in the soil, with a maximum exceedance factor of 31.5. The proportion of exceedance at our testing sites is 13.04%, mainly located in the coking area. Figure 2 shows the vertical distribution of BTEX in soil samples at the sampling points. The majority of soil samples with detectable BTEX are distributed at a depth of 1.5 m, accounting for 68.8% of all detected samples at different depths. The maximum concentrations of the five benzene components are located at a depth of 1.5 m in the AS07 sample site. Among them, benzene exceeded the screening value, with the highest detected content of 32.5 mg·kg^−1^. Yang et al. concluded that the migration and transformation of BTEX in the soil are significantly influenced by groundwater table fluctuation (GTF) [46]. This study shows similar results, where the detected BTEX are mainly found near the groundwater depth (1.5 m). The samples detected at this depth accounted for 68.8% of the total detected samples at different depths. Furthermore, the samples exceeding the standard were concentrated specifically at a depth of 1.5 m, indicating a close relationship between pollutant enrichment and the groundwater fluctuation zone. These results indicate that the benzene exceedance rate is high at soil sampling points, and the spatial distribution of benzene and toluene is extensive, with the pollution depth of benzene significantly influenced by the depth of groundwater.

#### 3.1.2. Pollution Characteristics and Spatial Distribution of BTEX in the Air

Pollutants in the air are influenced to some extent by meteorological conditions, photochemical reactivity, and other factors, leading to certain spatial distribution characteristics of pollutant types and concentrations in the soil and air. Table 3 shows the results for the quantitative analysis of BTEX present in the air at each sampling point. The BTEX were detected with average concentration levels as follows: toluene (4.59 μg·m^−3^) > *m*/*p*-xylene (2.92 μg·m^−3^) > benzene (1.86 μg·m^−3^) > ethylbenzene (1.47 μg·m^−3^) > *o*-xylene (1.37 μg·m^−3^). In this study, the total mass concentration of BTEX was obtained by summing up the different concentrations of various pollutants detected in the samples collected. The contribution rates of each component in the BTEX are shown in Table 3. Toluene is the major contributing pollutant in BTEX, detected in all sampling points, with a contribution rate of up to 72.58%. Benzene and ethylbenzene are the next major contributors, with detection rates of 39.13% and 47.83%, and contribution rates of 11.50% and 11.09%, respectively. Other components have lower detection rates and corresponding low contribution rates. At a regional scale, Liu et al. conducted a preliminary investigation on BTEX compounds in the atmosphere of rural areas in the North China Plain and found that the concentration ratio of benzene and toluene was high during the winter season [47]. Masih et al. conducted a year-long monitoring of BTEX in ambient air at a specific location in northern India and found the concentration order of BTEX to be toluene > benzene > ethylbenzene > xylene, with industrial emissions contributing the most [48]. At a smaller scale, Correa et al. collected 29 samples near a gas station in Brazil to assess the emission and dispersion of BTEX pollutants, and the results showed that the average concentration of toluene was as high as 47.7 μg·m^−3^, exceeding the concentrations of other components [49]. Bretón et al. found relatively high levels of toluene in the atmospheric pollutants near petroleum storage facilities in Mexico, especially during the dry season [50]. This study also found that toluene had the highest detected concentration of the pollutants, consistent with the conclusions drawn from previous studies. These results indicate that the sources of toluene are complex and widely present in the atmosphere of urban areas, rural areas, and polluted sites, with human activities and industrial production being the main emission pathways.

The research and monitoring of BTEX in the atmosphere has been conducted extensively in multiple countries and regions, and standards have been established for corresponding regional environmental air quality assessments. The United States first proposed a quality standard of 100 μg·m^−3^ for xylene in the Clean Air Act in 1990. In 2007, the Netherlands set a concentration standard of 10 μg·m^−3^ for benzene in the air. In 2010, the United Kingdom and Germany published annual average concentration limits of 5 μg·m^−3^ for benzene in their environmental air standards. In the same year, the European Union set target limits of 10 μg·m^−3^ for benzene and 25 μg·m^−3^ for toluene and xylene in its environmental quality standards [51,52,53,54]. At the moment, China has not set any specific evaluation standards for outdoor BTEX in the country’s environmental air quality standard. Although comprehensive emission standards and indoor air quality standards for BTEX were issued in 2014 and 2022, respectively [55,56], they do not align with the monitoring requirements of this site’s environmental air. The detected results in this study did not exceed the air quality standards established in foreign countries. However, the maximum concentration of benzene (4.98 μg·m^−3^) is close to the evaluation values (5 μg·m^−3^) in Germany, the United Kingdom, and the European Union, indicating that this pollutant requires special attention and that further control measures should be implemented.

**Figure 3:**
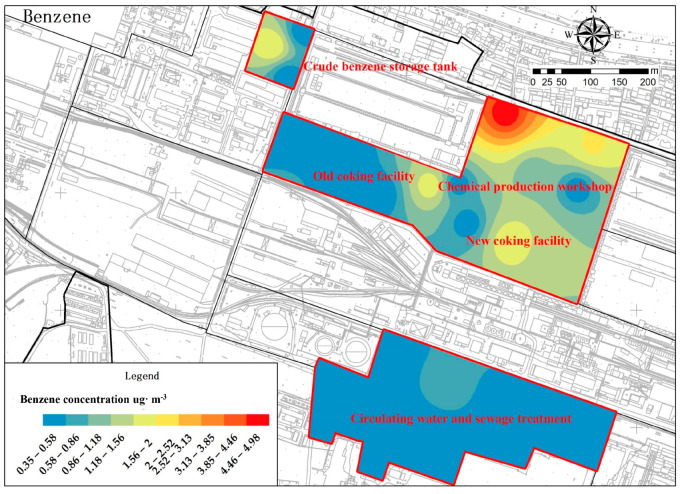

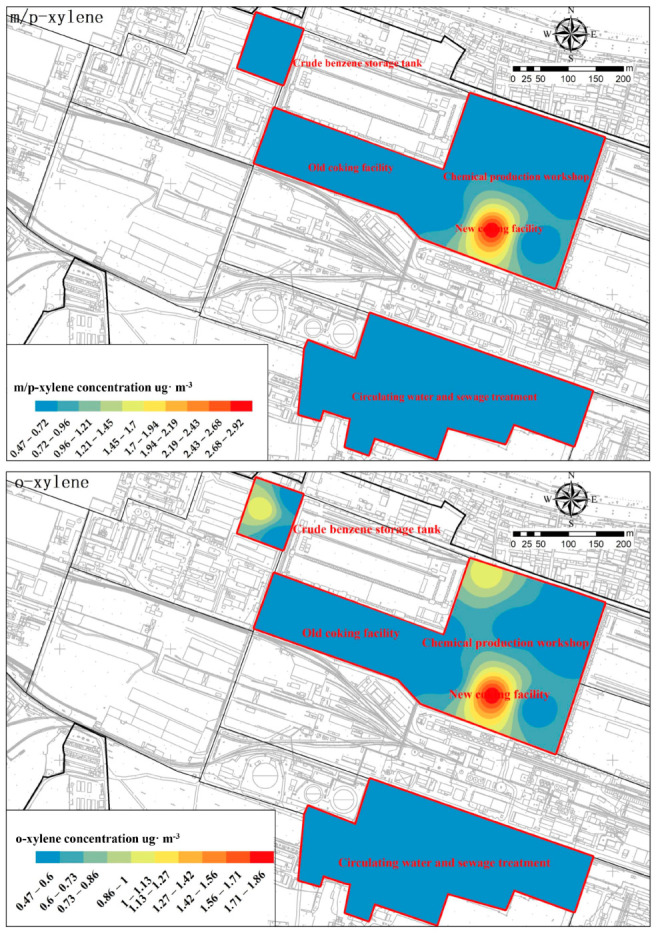

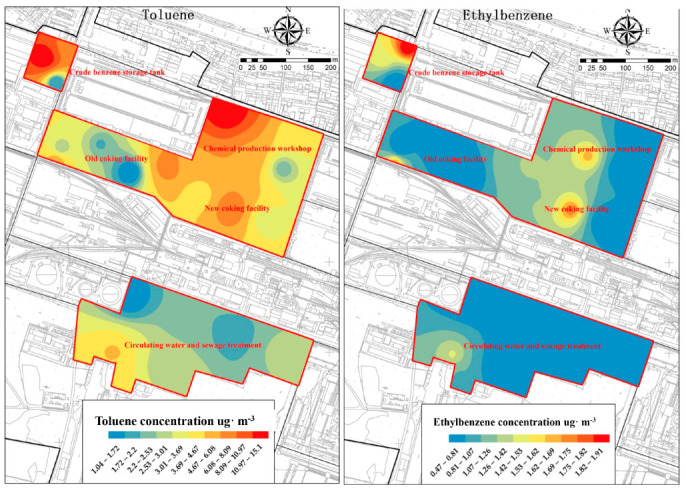
Spatial distribution characteristics of concentration for each component of BTEX.

Furthermore, through spatial interpolation and grid calculations, a detailed statistical analysis of the spatial distribution of BTEX at 23 monitoring points was conducted to reveal the spatial distribution characteristics of BTEX in the ambient air. Research has found that inverse distance weighting (IDW) is simpler and more direct to use than other methods, and is fast and accurate, especially for sites with fewer discrete points, and the maps form a circle around the high values, which gives a more intuitive representation of the pollutant concentration [57]. The concentrations of undetected components were calculated as half of the detection limit. From the grid shapes in Figure 3, it can be observed that they mainly appear in “point-like” and “area-like” patterns. *O*-xylene and *m*/*p*-xylene exhibit central features and have a larger coverage area for low concentrations. In terms of the number of high-value “spots” (benzene > 1.56 μg·m^−3^, toluene > 4.67 μg·m^−3^, ethylbenzene > 1.26 μg·m^−3^, *m*/*p*-xylene > 1.70 μg·m^−3^, *o*-xylene > 1.00 μg·m^−3^), *o*-xylene and *m*/*p*-xylene have fewer “spots”; three and one, respectively, mainly distributed in the coking area, showing similar spatial distribution. Toluene has as many as six high-value “spots”, distributed extensively in the coking area, chemical production workshops, and crude benzene storage areas. Benzene and ethylbenzene both have five high-value “spots” and share the same distribution areas. Looking at the spatial distribution of BTEX in the “spots” in each research area, the coking area has all five components, while the chemical production workshops and crude benzene storage area have four components. The circulating water and sewage treatment area only has two components, indicating that the coking area is the region with the highest emissions of BTEX. Studies have estimated that the BTEX exposure of workers in coal loading workshops and near the coking area may be higher than that of workers in other sections [58], confirming that coking is the main pollutant-generating process and BTEX are the dominant group of pollutants emitted and retained in the coking area.

### 3.2. Transport Flux of Pollutants in Soil and Air Phases

#### 3.2.1. Correlation Analysis of Soil–Air BTEX

The sources of BTEX in the atmosphere are subject to certain uncertainties. Previous studies have suggested that BTEX originates from vehicle exhaust emissions, chemical solvents, and fuel combustion [59,60]. Because the research areas are located in a typical polluted site, the transport patterns of BTEX as they are released from the soil into the atmosphere constitute an important pathway [20]. Existing research has shown that soil texture is one of the factors influencing the volatilization of BTEX in soil. The adsorption–desorption interactions between soil particles and organic carbon are important environmental chemical behaviors that directly affect the degradation, volatilization, and bioavailability of BTEX [61,62,63]. Pollutants in the atmosphere are somewhat correlated to meteorological conditions [64]. They can undergo oxidation and gas-particle distribution processes to form Secondary Organic Aerosols (SOA) and generate ozone based on reaction reactivity [65]. Therefore, there is a multitude of factors that could influence the actual monitoring data. As mentioned in Section 3.1, the detected concentrations of BTEX in the soil–air system are correlated. However, the corresponding relationship between concentrations in the soil and air media requires further correlation analysis.

The results of the correlation analysis of the BTEX in soil and air are shown in Table 4, indicating negative correlations for all components. Among them, *o*-xylene has the highest correlation coefficient at −0.839 (*p* < 0.01), followed by ethylbenzene and benzene, with excellent correlation coefficients of −0.809 (*p* < 0.01) and −0.554 (*p* < 0.05), respectively. The correlation between toluene and *m*/*p*-xylene is weaker, with *m*/*p*-xylene having the lowest correlation coefficient at 0.111, which is highly insignificant. In summary, this study primarily focuses on the transport flux and fate of benzene, ethylbenzene, and *o*-xylene as the three pollutant factors in the soil-air system.

#### 3.2.2. Transport of BTEX between the Soil and Air Phases

In response to the pollution characteristics of soil and air in the research areas, this research has selected three significant components (benzene, ethylbenzene, and *o*-xylene) among the soil-air BTEX. The transport flux at the soil-air interface for each component was calculated using the fugacity model.

According to the algorithm of the fugacity model, the direction of pollutant transport is determined by comparing the magnitudes of the air-phase fugacity (f_1_) and the soil-phase fugacity (f_3_). In order to describe this process more accurately, the concept of FE (fugacity entropy), f_3_/f_1_, was introduced. Determining the positive or negative value of logf_3_/f_1_ is of vital importance as it aids in the understanding of the transport trend of pollutants between the soil and air phases. Table 5 shows the transport fluxes between soil and air in four functional areas. Negative values of flux indicate that pollutants transport from air to soil, while positive values indicate transport from soil to air. Among the monitoring points, only AS11 has a negative fugacity entropy (−0.02), while the fugacity entropy values for other points are positive, ranging from 0.20 to 4.53. The proportions of benzene, ethylbenzene, and *o*-xylene transporting from soil to air are 90.9%, 100%, and 100%, respectively. The flux ranges for benzene, ethylbenzene, and *o*-xylene are 5-950,623 × 10^−7^, 14-191,970 × 10^−7^, and 13-219,122 × 10^−7^ mol·h^−1^, respectively. The contribution rates of these components are 60.55%, 12.43%, and 27.02%, respectively. The average transport fluxes of the components, from highest to lowest, are benzene (93,786 × 10^−7^ mol·h^−1^), *o*-xylene (62,786 × 10^−7^ mol·h^−1^), and ethylbenzene (16,507 × 10^−7^ mol·h^−1^). Cetin et al. studied the soil-air exchange law of PAHs in the soil of Turkey and Istanbul and found that low-molecular-weight pollutants were more likely to volatilize from soil to the atmosphere compared to high-molecular-weight ones [66]. In this study, benzene had the smallest molecular weight and the average transport flux was greater than the sum of *o*-xylene and ethylbenzene, which was consistent with the study of Cetin et al.

These results indicate that 94.1% of the monitoring points in the research areas show pollutants transporting from soil to air, while in the AS11 chemical production workshop, benzene transports from air to soil. Benzene is the dominant pollutant among the BTEX, contributing the most to the transport flux from soil to air. This suggests that benzene has a weaker adsorption capacity compared to the other compounds, while *o*-xylene and ethylbenzene are more widely present in soil and dust [67].

#### 3.2.3. Distribution Characteristics of Soil-Air BTEX Transport Flux

**Figure 4:**
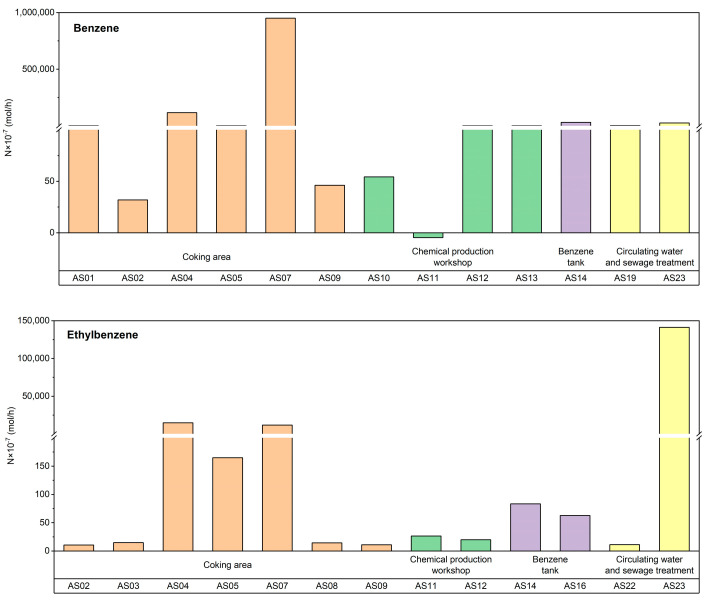

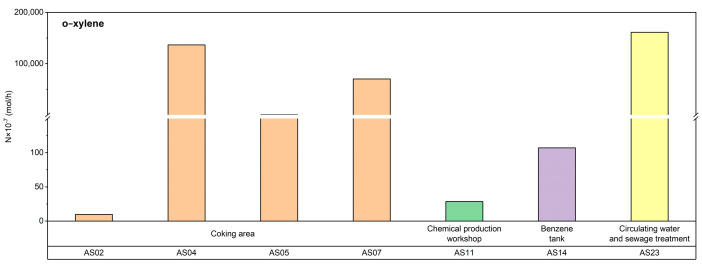
Transport Flux of BTEX in Soil-Air Media.

The transport flux of BTEX is somewhat related to human activities and functional zones. Based on the data from Figure 4, it is possible to determine the average transport flux order of benzene, ethylbenzene, and *o*-xylene in each research area. Among them, the order of average benzene transport flux is: coking area (178,030 × 10^−7^ mol·h^−1^) > crude benzene storage area (30,500 × 10^−7^ mol·h^−1^) > circulating water and sewage treatment area (12,955 × 10^−7^ mol·h^−1^) > chemical production workshop (281 × 10^−7^ mol·h^−1^). The average transport fluxes of ethylbenzene and *o*-xylene are highest in the circulating water and sewage treatment area, with values of 70,623 × 10^−7^ mol·h^−1^ and 161,213 × 10^−7^ mol·h^−1^, respectively, while the chemical production workshop has the lowest average transport flux. It is therefore obvious that there are significant differences in transport fluxes among different research areas. The benzene transport flux in the coking area is dominant, indicating a larger emission of benzene from soil to air in that area. The main controlling factors affecting the transport flux variations in the circulating water and sewage treatment area are ethylbenzene and *o*-xylene, as these pollutants have higher emissions from soil to air. Previous studies have indicated that the areas with the highest emission factors for BTEX during coal coking processes are coke ovens, coke discharging area, and sewage treatment area [58,68]. These results suggest that the coking area and sewage treatment area are key areas where BTEX are highly prone to accumulate in the soil in the steel plant. Considering the emission characteristics of pollutants in different research areas, appropriate remediation and control measures need to be implemented for the site.

### 3.3. Health Risk Assessment for BTEX

#### 3.3.1. Health Risks of BTEX in the Soil

**Figure 5:**
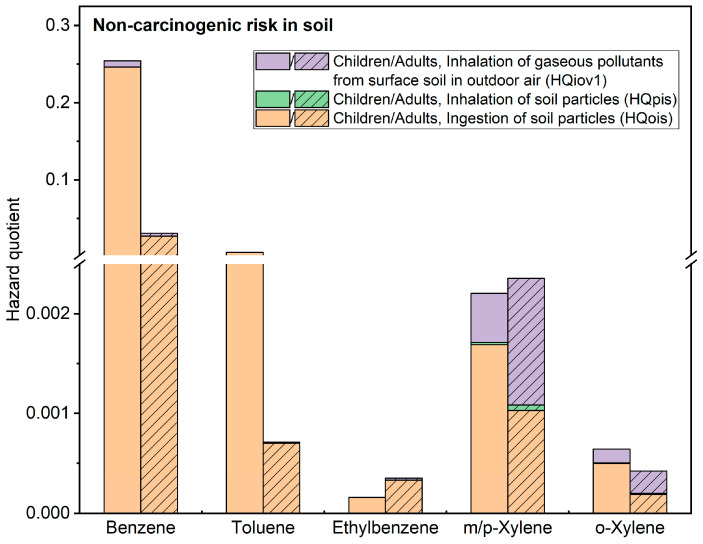
Non-carcinogenic Health Risk Assessment Results for BTEX under Different Exposure Pathways.

This study establishes a connection between soil BTEX pollution and human health, using the level of health risk as an evaluation indicator. Human health risks are closely related to parameters such as pollutant toxicity, soil physicochemical properties, and exposed receptors. Figure 5 presents the results of the non-carcinogenic health risk assessment of BTEX in both children and adults, in which three exposure pathways are considered. HIn represents the total non-carcinogenic risk index caused by BTEX through three exposure pathways, i.e., the sum of HQois, HQpis, and HQiov1. Different BTEX have different pathways of non-carcinogenic health risks. The relative magnitude of harm from BTEX exposure pathways is HQois > HQiov1 > HQpis. Moreover, the pathway’s impact on children and adults is consistent in the area, with ingestion of soil particles being the main pathway for both children and adults exposed to contaminated soil. For children’s non-carcinogenic risks, the order of HIn from highest to lowest is: benzene (2.54 × 10^−1^) > toluene (6.34 × 10^−3^) > *m*/*p*-xylene (2.21 × 10^−3^) > *o*-xylene (6.42 × 10^−4^) > ethylbenzene (1.61 × 10^−4^). Although the non-carcinogenic risk values for each component of BTEX are below the acceptable risk threshold (HQ < 1), the maximum non-carcinogenic risk value for benzene is close to 1, indicating that the risk of this pollutant should not be ignored. Among all sampling points, the range of non-carcinogenic risk values for benzene and toluene under the children’s exposure scenario is 4.63 × 10^−8^~2.46 × 10^−1^ and 2.67 × 10^−10^~6.32 × 10^−3^, respectively. The range of non-carcinogenic risk values under the adult exposure scenario is 1.99 × 10^−8^~2.73 × 10^−2^ for benzene and 1.15 × 10^−10^~7.01 × 10^−4^ for toluene. The maximum risk values differ by an order of magnitude between the two scenarios.

**Figure 6:**
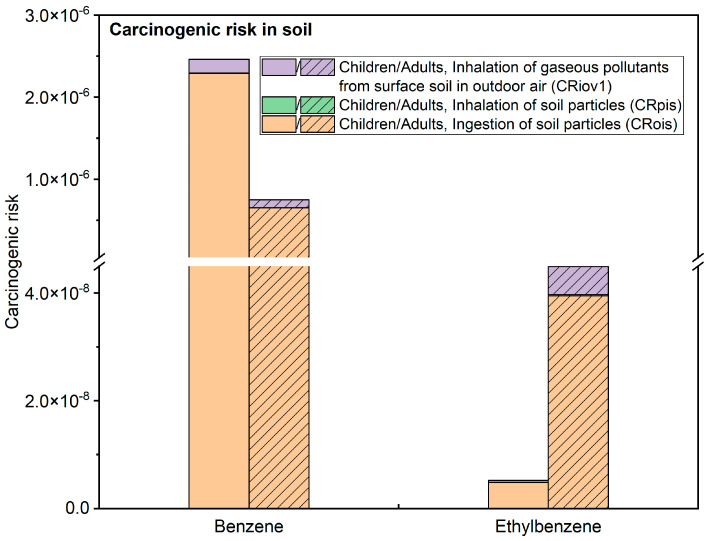
Carcinogenic Health Risk Assessment Results for BTEX under Different Exposure Pathways.

Figure 6 shows the carcinogenic risk assessment results of soil BTEX for children and adults in this study. Among the BTEX, only benzene and ethylbenzene have been calculated for their carcinogenic risk levels. The harm level of exposure pathways for soil BTEX in the study area is CRois > CRiov1 > CRpis. Among them, ingestion of soil particles is the main pathway for the carcinogenicity of BTEX, contributing to 93.1% for children and 87.0% for adults in terms of carcinogenic risks. The total carcinogenic risk values for benzene and ethylbenzene in the children’s exposure scenario are 2.46 × 10^−6^ and 5.22 × 10^−9^, respectively, while in the adult’s exposure scenario, the total carcinogenic risk values are 7.50 × 10^−7^ and 4.49 × 10^−8^, respectively. It should be noted that the total carcinogenic risk value for benzene in the children’s exposure scenario exceeds the carcinogenic risk threshold of 1 × 10^−6^. The range of carcinogenic risk values across all sampling points is between 9.61 × 10^−13^ and 2.29 × 10^−6^, with 12.5% of the points exceeding the acceptable carcinogenic risk level. However, the carcinogenic risk levels in the adult exposure scenario are acceptable.

The health risks associated with BTEX in soil are widespread. In the study by Xia [69], two typical petroleum-contaminated sites were selected, and its results showed that both the carcinogenic and non-carcinogenic risks of BTEX in soil exceeded the target level. These findings indicate that this study is similar to pollution sites in the petroleum industry, where BTEX, as characteristic pollutants, pose significant health risks to the human body, especially for children who are more susceptible to harm in their daily lives. Therefore, it is crucial to implement proper land planning and utilization in the later stages of the study area. Strict control measures should be implemented to mitigate the presence of benzene in soil.

#### 3.3.2. Health Risks of BTEX in the Air

**Figure 7:**
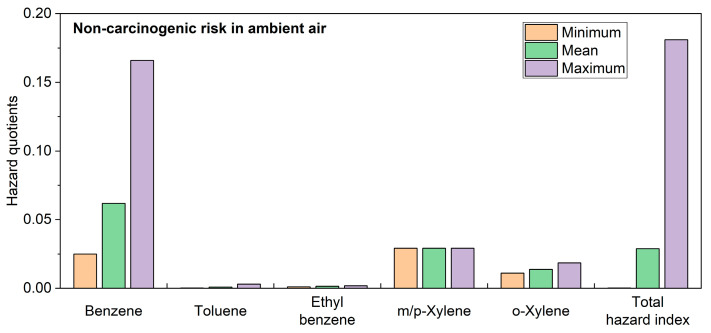

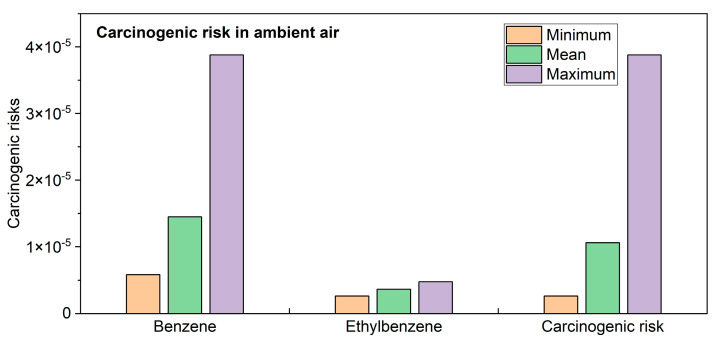
The Level of Human Health Risk Posed by BTEX in Ambient Air.

Long-term exposure to airborne BTEX can irritate the skin and mucous membranes and cause damage to the major systems of the human body. This study assessed the health risks of exposure to air pollutants, the results of which are shown in Figure 7. The hazard quotients (non-carcinogenic risks) for different components of BTEX ranged from 1.10 × 10^−2^ to 1.66 × 10^−1^, with benzene having the highest hazard quotient, reaching a maximum value of 1.66 × 10^−1^, and the non-carcinogenic average level of toluene being the lowest. During the study period, the fluctuation range of the total HI was between 2.08 × 10^−4^ and 1.81 × 10^−1^, with HI values at all sampling points not exceeding the safety threshold of 1, indicating acceptable levels of non-carcinogenic risks.

The carcinogenic risk range of each component in the air stood between 2.63 × 10^−6^ and 3.88 × 10^−5^. All sampling points in the study area exhibited acceptable carcinogenic risks above the threshold of 10^−6^. The range of carcinogenic risk values for benzene and ethylbenzene was 5.85 × 10^−6^~3.88 × 10^−5^ and 2.63 × 10^−6^~4.78 × 10^−6^, respectively, with the average level of carcinogenic risk being higher for benzene (1.45 × 10^−5^) compared to ethylbenzene (3.66 × 10^−6^). Based on the aforementioned subdivision of carcinogenic risk into four levels, benzene exceeded the safety threshold, indicating a high probability of risk (1 × 10^−5^ < Risk < 1 × 10^−4^), which means that there is a substantial health threat to individuals in case of long-term exposure. Ethylbenzene presented a low probability of risk (1 × 10^−6^ < Risk < 1 × 10^−5^), but its health risks should not be ignored. Among the study points, 28.6% had a high probability of risk, while the remaining sites had a low probability of risk.

Research on the health risks of airborne BTEX has become a hot topic in the academic circle. Studies from regions like South Africa and Turkey indicate that the health risks posed by exposure to BTEX are all within acceptable levels [70,71]; Hedayatzade et al. discovered a non-carcinogenic risk value of 5.75 for BTEX in the Ahvaz region of Iran, which is significantly higher than the standard value of 1, and the carcinogenic risk levels of benzene are considered acceptable [72]. Khoshakhlagh et al. conducted a health risk assessment for BTEX in a composite manufacturing plant [73]. The results showed that the non-carcinogenic risk values for benzene, ethylbenzene and xylene were 46.00, 6.96, and 22.4 times higher than the threshold set by the USEPA. These findings indicate that BTEX originates from diverse sources and poses a certain level of harm to human health in certain regions. Therefore, for the decommissioned site studied in this research, institutional controls can be implemented to reduce the exposure time (ET) and exposure frequency (EF), decrease the exposure concentration, and thus lower the carcinogenic risks.

**Figure 8:**
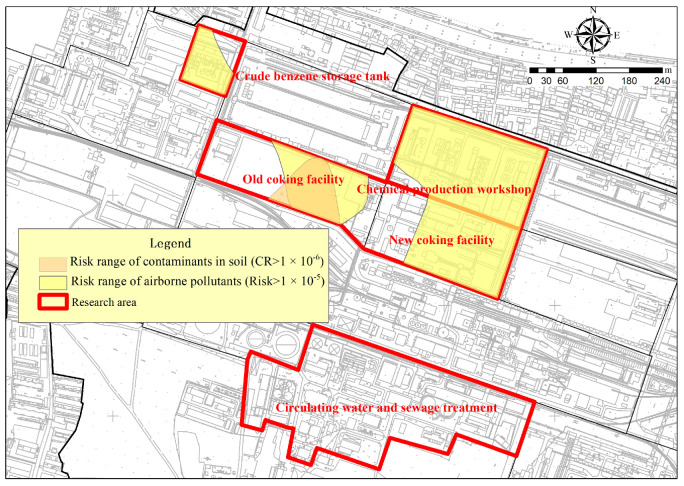
Risk areas for soil and airborne contaminants.

This study examined the health risks associated with soil and ambient air exposure. The historic coking area is the risk zone where both soil and air quality must be considered simultaneously, as depicted in Figure 8. Neither media posed non-carcinogenic risks to the exposed population. The main pathway contributing to the carcinogenic risk from benzene in soil was through oral ingestion of soil particles, with a maximum carcinogenic risk value of 2.29 × 10^−6^. In the same pathway, the maximum carcinogenic risk value for the inhalation of pollutants from surface soil in the air was 1.66 × 10^−7^, while the direct inhalation of ambient air resulted in a maximum carcinogenic risk value of 3.88 × 10^−5^. The difference between the two pathways is nearly 234-fold, indicating a higher carcinogenic risk from ambient air exposure. This may be due to the more conservative health assessment model parameters used for ambient air; the stricter calculation of exposure time (ET) and exposure frequency (EF), which is year-round and all-day; as well as the wider range of pollutant sources [60]. Therefore, the actual monitored concentrations may be higher than those released from the surface soil.

## 4. Conclusions and Recommendations

(1) Both soil and air at the study sites show significant benzene pollution. The maximum exceedance factor for benzene in soil samples is 31.5, accounting for 13.04% of the total sampling points, with the highest concentration occurring at the depth of the capillary zone. The maximum concentration of benzene in air samples is 4.98 μg·m^−3^, which is close to the evaluation standards in Germany, the UK, and the EU, and the probability of exceeding the standard is high among the pollutants of concern.
(2) The correlation analysis of BTEX indicates that *o*-xylene has the strongest correlation coefficient of −0.839 (*p* < 0.01), followed by ethylbenzene and benzene, which also show excellent correlation; there is a certain degree of exchange between these three components in the soil–air medium.
(3) The results from the multi-media fugacity model show that 94.1% of sampling points show a trend of transport from the soil phase to the air phase. The average transport flux levels from highest to lowest are benzene (93,786 × 10^−7^ mol·h^−1^) > *o*-xylene (62,786 × 10^−7^ mol·h^−1^) > ethylbenzene (16,507 × 10^−7^ mol·h^−1^). Benzene is the pollutant that contributes the most to the transport flux from soil to air within the site.
(4) The BTEX in both the soil and air at the study sites can pose health risks to humans. The non-carcinogenic risk values for BTEX in soil are below the acceptable risk level (HQ < 1). The total carcinogenic risk value for benzene in children’s exposure scenarios exceeds the carcinogenic risk level (1 × 10^−6^), and the proportion of sampling points exceeding the acceptable carcinogenic risk level is 12.5%. The fluctuation range of the hazard index (HI) for BTEX in ambient air is between 2.08 × 10^−4^ and 1.81 × 10^−1^, and all sampling points have HI values below the safety threshold of 1. The range of the carcinogenic risk values for BTEX in the air is between 2.63 × 10^−6^ and 3.88 × 10^−5^, and all sampling points in the research area have acceptable carcinogenic risks exceeding the threshold of 10^−6^. Through institutional control, managers can shorten the ET and EF of the population, reduce the exposure concentration, and thus reduce the risk of cancer.
(5) Our proposal aims to decrease the duration and frequency of population exposure by implementing institutional control measures. This approach seeks to lower the concentration of exposure and mitigate the risk of carcinogenesis. In locations designated for coking and sewage treatment, where the potential for benzene accumulation is significant, it is advisable to employ in situ oxidation or in situ thermal desorption techniques for soil remediation, particularly where the intended land use in the future is residential. These methods allow for the treatment of contaminated soil without the need for excavation or disturbance.

## Figures and Tables

**Table 1:** Actual parameter values for exposure assessment.

Parameter	Parameter Name	Unit	Value
d	Thickness of contaminated topsoil layer	cm	150
f_om_	Soil organic matter content	g·kg^−1^	19
ρ_b_	Soil bulk density	kg·dm^−3^	1.64
P_ws_	Soil moisture content	kg·kg^−1^	0.223
ρ_s_	Soil particle density	kg·dm^−3^	2.7
PM_10_	Concentration of inhalable particulate matter in the air	mg·m^−3^	0.08

**Table 2:** Analysis of BTEX detection results in soil samples.

Pollutant Component	Minimum Value (mg·kg^−1^)	Average Value (mg·kg^−1^)	Maximum Value (mg·kg^−1^)	Detection Rate	Maximum Exceedance Multiple over Screening Value *	Proportion of Sites in Exceedance
Benzene	0.0066	2.69	32.51	30.43%	31.5	13.04%
Toluene	0.0063	1.81	16.70	30.43%	-	-
Ethylbenzene	0.0077	3.39	9.84	13.04%	-	-
*m*/*p*-xylene	0.0104	14.11	61.50	21.74%	-	-
*o*-xylene	0.0183	4.79	11.20	13.04%	-	-

Note: * Refers to the screening values for Class I land and the formula provided in “Soil Environmental Quality—Risk Control Standard for Soil Contamination of Development Land (Trial)” (GB 36600-2018) and “Technical Guidelines for Risk Assessment of Soil Contamination of Land for Construction” (HJ 25.3-2019), respectively.

**Table 3:** Concentration levels of BTEX detected in the air (Unit: μg·m^−3^).

Pollutant Component	Limit of Detection	Minimum Value	Maximum Value	Average Value	Standard Deviation	Number of Detections	Detection Rate	Contribution Rate
Benzene	0.7	0.75	4.98	1.86	1.30	9	39.13%	11.50%
Toluene	0.82	1.04	15.1	4.59	3.67	23	100.00%	72.58%
Ethylbenzene	0.95	1.05	1.91	1.47	0.27	11	47.83%	11.09%
*m*/*p*-xylene	0.95	2.92	2.92	2.92	-	1	4.35%	2.01%
*o*-xylene	0.95	1.1	1.86	1.37	0.43	3	13.04%	2.82%

**Table 4:** Correlation Analysis of Soil-Air BTEX.

Pollutants Concerned	Correlation Coefficient	Significance (Two-Tailed)
Benzene	−0.554 *	0.04
Toluene	−0.111	0.606
Ethylbenzene	−0.809 **	0
*m*/*p*-xylene	−0.577	0.134
*o*-xylene	−0.839 **	0.009

Note: *. Highly significant correlation at the 0.05 level (Two-tailed); **. Highly significant correlation at the 0.01 level (Two-tailed).

**Table 5:** Soil-Air Transport Flux of BTEX at Each Sampling Point in the Research Areas.

Sampling Point No.	Research Area	Benzene		Ethylbenzene		*o*-Xylene	
		FE (Dimensionless)	N (1 × 10^−7^ mol·h^−1^)	FE (Dimensionless)	N (1 × 10^−7^ mol·h^−1^)	FE (Dimensionless)	N (1 × 10^−7^ mol·h^−1^)
AS01	Coking Area	1.80	940				
AS02		0.38	32	0.23	11	0.21	10
AS03				0.38	15		
AS04		4.46	115,907	3.56	14,736	4.53	136,602
AS05		1.48	635	1.22	165	2.00	396
AS07		5.37	950,623	3.47	11,847	4.24	70,287
AS08				0.36	14		
AS09		0.80	46	0.24	11		
AS10	Chemical Production Workshop	0.36	54				
AS11		−0.02	−5	0.38	26	0.44	29
AS12		1.38	410	0.25	20		
AS13		1.73	378				
AS14	Crude Benzene Storage Area	2.28	30,500	0.29	83	0.42	107
AS16				0.20	63		
AS19	Circulating Water and Sewage Treatment Area	2.20	1222				
AS22				1.94	11		
AS23		3.86	24,688	4.27	141,234	4.67	161,213

## Data Availability

The data presented in this study are available on request from the corresponding author. The data are not publicly available due to confidentiality.

## Supplementary Materials

The following supporting information can be downloaded at: https://www.mdpi.com/article/10.3390/toxics11100868/s1, Section S1. Construction of the Fugacity Model [35,36,37,39]; Section S2. Human Health Risk Assessment Methods.
