# Supplementary material for: An Analysis of the Spatial Characteristics and Transport Fluxes of BTEX in Soil and Atmospheric Phases at a Decommissioned Steel Mill Site in China with a Long History
Source: Toxics. 2023 Oct 18;11(10):868. doi: 10.3390/toxics11100868 (PMC10610661; doi:10.3390/toxics11100868)
Supplement: Supplementary file 1 [file toxics-11-00868-s001.zip › toxics-2659539-supplementary.pdf]

toxics

Supplementary Materials: An Analysis of the Spatial Characteristics and Transport Fluxes of BTEX in Soil and Atmospheric Phases at a Decommissioned Steel Mill Site in China with a Long History

Xuwei Li, Wenyi Xie, Da Ding, Mengjie Wang, Lingya Kong, Dengdeng Jiang and Shaopo Deng

1. Construction of the Fugacity Model

Table S1. Main physicochemical parameters of the target compounds [35,36].

Name of substance	$M(g·mol^{-1})$	K_{OW}	$P_L(Pa)$	$P_{LS}(Pa)$	$S(mol·m^{-3})$
Benzene	78.11	134.90	12700	3535	0.022788
Ethylbenzene	106.17	1412.54	1352	147	0.001592
o-Xylene	106.17	1318.26	885	172	0.001677

Note: M is the molecular mass; P_L is the vapor pressure at 25℃; P_{LS} is the vapor pressure of super-cooled liquid at 0℃; S is the solubility at 25℃.

Table S2. Environmental parameters of the study area[37].

Parameters	Atmosphere	Soil
$R(Pa·m^3·mol^{-1}·K^{-1})$	8.314	-
T(K)	298	-
V_{13}	1.04E-10	-
V_{31}	-	0.2
V_{32}	-	0.3
V_{33}	-	0.5
y_{oc}*	-	0.011
ρ_s*($kg·m^{-3}$)	-	2.01

Note: * is the actual test value; R is the gas constant; T is the temperature; V_{13} is the volume fraction of particulate matter in the air; V_{31} is the volume fraction of air in the soil; V_{32} is the volume fraction of moisture in the soil; V_{33} is the volume fraction of soil particles in the soil; y_{OC} is the mass fraction of organic carbon in the soil; and ρ_S is the density of the soil.

Table S3. Migration parameters in the study area [37].

Parameters	Notation	Numerical value	Unit
Gas-side mass transfer coefficient at the soil/gas interface	K_{SA}	1.00	$m·h^{-1}$
Diffusion path length in soil	Y_3	0.05	
Molecular diffusion coefficient in air	B_{A3}	0.04	$m^2·h^{-1}$
Molecular diffusion coefficient in water	B_{W3}	0.000004	$m^2·h^{-1}$
Clearance	Q	200000	
Air-soil exchange area *	A_{13}	Size of the study areas	m^2
Air-water exchange area *	A_{12}	Size of the study areas	m^2
Precipitation rate *	U_Q	0.00011283	$m·h^{-1}$
Dry deposition rate	U_P	10.8	$m·h^{-1}$

Note: * are actual test values.

2. Human Health Risk Assessment Methods [39]

2.1. Calculation of exposure by different routes

(1) Oral route of soil ingestion

Carcinogenic risk formula:

$$OISERca = \frac{\left(\frac{OSIRc \times EDc \times EFc}{BWc} + \frac{OSIRa \times EDa \times EFa}{BWa}\right) \times ABSo}{ATca} \times 10^{-6}$$

Non-cancer risk formula:

$$OISERnc = \frac{OSIRc \times EDc \times EFc \times ABSo}{BWc \times ATnc} \times 10^{-6}$$

(2) Dermal route of exposure to soil

Carcinogenic risk formula:

$$DCSERca = \frac{SAEc \times SSARc \times EFc \times EDc \times Ev \times ABSd}{BWc \times ATca} \times 10^{-6} + \frac{SAEa \times SSARa \times EFa \times EDa \times Ev \times ABSd}{BWa \times ATca} \times 10^{-6}$$

Carcinogenic risk formula:

$$DCSERnc = \frac{SAEc \times SSARc \times EFc \times EDc \times Ev \times ABSd}{BWc \times ATnc} \times 10^{-6}$$

(3) Exposure routes for inhaled soil particles

Carcinogenic risk formula:

$$PISERca = \frac{PM10 \times DAIRc \times EDc \times PIAF \times (fspo \times EFOc + fspi \times EFIc)}{BWc \times ATca} \times 10^{-6} + \frac{PM10 \times DAIRa \times EDa \times PIAF \times (fspo \times EFOa + fspi \times EFIa)}{BWa \times ATca} \times 10^{-6}$$

Carcinogenic risk formula:

$$PISERnc = \frac{PM10 \times DAIRc \times EDc \times PIAF \times (fspo \times EFOc + fspi \times EFIc)}{BWc \times ATnc} \times 10^{-6}$$

(4) Exposure pathways for volatile contaminants from inhalation of outdoor surface soils

Carcinogenic risk formula:

$$IOVERca1 = VFsuroa \times \left(\frac{DAIRc \times EFOc \times EDc}{BWc \times ATca} + \frac{DAIRa \times EFOa \times EDa}{BWa \times ATca}\right)$$

Carcinogenic risk formula:

$$IOVERnc1 = VFsuroa \times \frac{DAIRc \times EFOc \times EDc}{BWc \times ATnc}$$

Where: the subscript c represents children and a represents adults; $OISER_{ca}$, $DCSER_{ca}$, $PISER_{ca}$ and $IOVER_{ca1}$ represent soil exposures from oral ingestion, dermal contact, inhalation of soil particulate matter, and inhalation of volatile contaminants in outdoor surface soil, respectively, kg soil·kg^{-1} weight·d^{-1}; ABSo and ABSd are absorption efficiency factors for oral and dermal uptake, respectively, dimensionless and taken as 1; CRois, CRdcs and CRpis represent carcinogenic risks from the oral soil ingestion pathway, dermal soil contact pathway, and inhalation of soil particulate matter pathway, dimensionless; Csur is the contaminant content in topsoil, μg·g^{-1}; SER is the percentage of exposed skin, dimensionless; C(PM10) is the airborne PM mass concentration, mg·m^{-3}, taken as the annual average mass concentration in the region in 2021, 0.062 mg·m^{-3}.

2.2. Calculation of carcinogenicity risk and hazard quotient

CR= Exposure by exposure route × C_{sur} × Carcinogenic Slope Factors or Factors by Exposure Pathway

HQ= Exposure by exposure route × C_{sur} / Reference dose by exposure route × SAF

Where: CR is the carcinogenic risk of the contaminant; HQ is the hazard quotient of the contaminant; C_{sur} is the concentration of the contaminant in the surface soil, mg·kg^{-1}; SAF is the reference dose partition coefficient for exposure to soil.

References

35. Linstorm, P. Nist chemistry webbook, nist standard reference database number 69. *J. Chem. Eng. Data* **1998**, *9*, 1059–1063. https://doi.org/10.1021/je000236i.
36. Haynes, W.M. *CRC Handbook of Chemistry and Physics*; CRC Press: Boca Raton, FL, USA, 2014. Available online: https://analysischamp.com/CRCHandbook01.pdf (accessed on 1 June 2023).
37. Parnis, J.M.; Mackay, D. *Multimedia Environmental Models: The Fugacity Approach*; CRC press: Boca Raton, FL, USA, 2020. https://doi.org/10.1201/9781420032543.
39. *HJ 25.3-2019*; Technical Guidelines for Risk Assessment of Soil Contamination of Land for Construction. China Environmental Science Press: Beijing, China, 2019. Available online: https://www.mee.gov.cn/ywgz/fgbz/bz/bzwb/trhj/201912/t20191224_749893.shtml (accessed on June 2023).
